# Differences and similarities in outpatient antibiotic prescribing among advanced practice clinicians and physicians—Where do we go from here?

**DOI:** 10.1017/ice.2023.217

**Published:** 2024-01

**Authors:** Carissa M. Windish, Priya Nori

**Affiliations:** Division of Infectious Diseases, Department of Medicine, Montefiore Health System, Albert Einstein College of Medicine, Bronx, New York

Curbing inappropriate antibiotic use in the outpatient setting is both a major challenge and opportunity for antimicrobial stewardship. One of the barriers to effective intervention in this setting is the heterogeneity of providers with diverse prescribing practices.^1^ Characterizing antibiotic prescribing patterns by provider type is a starting point to developing targeted ambulatory stewardship interventions, and prior results show that advanced practice clinicians (APCs) are more likely to prescribe antibiotics for syndromes contributing to the heaviest excesses in outpatient antibiotic consumption compared to physicians.^1–4^ In the recent study “Comparison of antibiotic prescribing between physicians and advanced practice clinicians,” Hersh et al.^5^ confirmed these findings. Redemonstration of this pattern highlights the need for a focus shift; understanding the reason for these differences such that targeted solutions can be designed is of utmost importance.

Hersh et al. compared antibiotic prescribing patterns between physicians and APCs using a national sample of outpatient visits from 2010 to 2018. This retrospective study of ambulatory prescriptions for acute respiratory tract infections (ARTIs), including acute otitis media, sinusitis, pharyngitis or tonsillitis, evaluates the differences in overall and first-line antibiotic prescribing (when indicated), stratified by provider type (ie, physician or APC).^5^

Data were obtained from the National Ambulatory Medical Care Survey and the National Hospital Ambulatory Medical Care Survey, which are conducted annually by the National Center for Health Statistics. For ARTI visits, diagnoses were grouped into 3 tiers according to whether antibiotics were almost always indicated (tier 1), sometimes indicated (tier 2), or rarely indicated (tier 3).^5^ The proportion of visits resulting in an antibiotic prescription were compared by provider type associated with the visit. For visits with a diagnosis of acute otitis media, sinusitis, or pharyngitis and tonsillitis, the proportion of visits yielding a prescription for a first-line antibiotic was assessed. The proportion of visits involving a patient with a chronic condition were also compared by provider type to assess possible case-mix differences.^5^

Overall, 58% of APC-associated visits resulted in antibiotic prescriptions, compared to 52% of physician-associated visits. In subgroup analyses by age, setting, and diagnosis tier, there was a statistically significantly higher percentage of prescriptions written by APCs for children, in office settings, and for tier 2 diagnoses.^5^ Multivariate analysis showed higher rates of antibiotic prescribing for APC-associated ARTI visits with tier 2 or 3 diagnoses, which suggests that APCs inappropriately prescribe antibiotics for ARTIs more frequently than physicians.^5^ These results are consistent with prior work.^1–4^

The reason for this discrepancy is unclear, but the researchers hypothesize that case-mix differences (though not observed in this study), varying educational emphasis on antimicrobial stewardship during training and in continuing education, and lack of APC representation in stewardship leadership roles are potential contributing factors.^5^ Because the number of APCs comprising the clinician workforce continues to grow,^6^ we need to better understand how antibiotic stewardship efforts in the outpatient setting can best be designed to incorporate APCs.

Prescribing patterns between APCs and physicians were similar regarding first-line antibiotic choice; however, both groups performed poorly. Results indicate a lack of first-line antibiotic selection for ∼50% of ARTI visits, consistent across all provider types, reinforcing the need for enhanced stewardship educational efforts for all provider specialties.^5^

Study findings may not reflect antibiotic prescribing in all outpatient settings. Methods limit the ability to evaluate antibiotic prescribing patterns in ambulatory practices without physician oversight; thus, the frequency of inappropriate antibiotic prescribing for ARTIs by independently practicing APCs could potentially be higher than is reported in this study.^5^ Other limitations include lack of inclusion of urgent care and retail centers, which are sites that have been shown to exhibit higher rates of antibiotic prescribing than other outpatient centers^7^ and which may also have resulted in a lower frequency of inappropriate antibiotic use reported compared to all outpatient settings. Also unknown is the volume of visits at each setting that may have provided insight into the role of time pressures and excess patient volume on antibiotic prescribing rates. Rates of prescriptions for other types of medications to treat ARTI (eg, decongestants, cough suppressants, inhalers, etc) between physicians and APCs were also not explored in this study. Lastly, the impact of geographic location, patient insurance, socioeconomic status, race, ethnicity, and other social determinants of health on inappropriate antibiotic prescription is a priority area of study to understand how these factors influence our behaviors as clinicians. For example, Goyal et al.^8^ showed higher rates of inappropriate prescribing among non-Hispanic White children. Additional studies are needed to explore this phenomenon more deeply.

These findings add to the growing body of evidence demonstrating the need to develop adaptable outpatient antimicrobial stewardship interventions as the workforce composition changes. Hersh et al.^5^ have identified important starting points such as partnering with APC professional societies to develop education materials, to augment the role of antimicrobial stewardship in APC professional society meetings, and to promote APC involvement in stewardship leadership and development. Approaches should also encourage practices to employ techniques that have been shown to be effective in reducing the frequency of antibiotic prescribing, included provider-facing dashboards with peer comparisons, as well as more intensive education at practice meetings.^9^

More research is needed to elucidate why and in which specific ways prescribing practices differ between physicians and APCs, such that effective interventions can be crafted appropriately. Future research should focus on expanding the breadth of settings evaluated, including urgent-care centers, retail clinics, dental practices, etc, exploring the reasons for these differences, and evaluating the effectiveness of proposed interventions.
